# Knowledge and handling practices for raw hen's eggs during purchase, preparation, storage, and consumption: a cross sectional study

**DOI:** 10.1038/s41598-024-56288-8

**Published:** 2024-03-26

**Authors:** Mohammed Sabbah, Kamal Badrasawi, Lubna Kharraz, Manal Badrasawi

**Affiliations:** 1 Department of Nutrition and Food Technology, An-Najah National University, Nablus, Palestine; 2 College of Humanities and Educational Sciences, Palestine Polytechnic University, Hebron, Palestine; 3 Medicine and Health Science College, An-Najah National University, Nablus, Palestine

**Keywords:** Public health, Consumer behavior, Hen’s eggs consumption, Consumer knowledge, Palestine, Nutrition, Public health

## Abstract

The study aimed to assess the hen’s eggs knowledge, handling practices, and consumption among Palestinian consumers using the cross-sectional survey design. Online questionnaire was sent via social media platforms including well-known Facebook and WhatsApp groups in West bank, Gaza and Jerusalem, along with an invitation explaining the research objectives and the voluntary participation. The online questionnaire consisted of three main sections: socio demographic, knowledge and practices for hen’s eggs during purchase, preparation, storage, and consumption. The items were developed based on previous literature and international guidelines for hen's eggs purchase and handling, and subjected to content validity process, followed by a pilot study to determine the reliability of the questionnaire using Cronbach’s alpha test. The final data were analysed based on the dichotomous Rach model for knowledge and polytomous Rasch model for practices using Winsteps version 5.1.3. The Rasch SPSS output interval data files were used in the inferential analyses tests (one-way ANOVA test and independent samples t-test). The Rasch analysis showed that on average the participants had quite low level of knowledge of hen's eggs consumption safety, (person ability Mean 0.10 logit). The item difficulty measures ranged between + 1.65 (most difficult) and − 2.24 logit (easiest). It was not easy for the participants to endorse the items pertaining their practices in handling the hen’s eggs during purchase, preparation, storage and consumption, (person ability Mean − 0.11 logit). The item difficulty measures ranged between + 2.68 logits (most difficult) to − 2.45 logit (easiest). In addition, female participants significantly outperformed males in knowledge scores; and the level of hen's eggs hygiene practices and storage during purchase depended on participants’ knowledge level. The research recommended interventional programs to enhance Palestinians’ awareness and knowledge about hen's eggs knowledge, handling practices, and consumption. Further quantitative and qualitative research studies were also recommended.

## Introduction

In a time when hen’s eggs are a gourmet joy in many different types of cuisine and a morning staple, have you ever pondered about the subtleties involved in guaranteeing the safety and quality of this seemingly common food item?. Hen's eggs are a low-cost food item that is widely consumed on a global scale^1,2^. In recent years, global hen's eggs production and consumption have increased in lockstep with rising demand for hen's eggs and animal proteins^3^. Numerous intrinsic product characteristics, such as yolk color, hen's eggs shell color, size, and appearance, as well as extrinsic product characteristics, such as size, freshness, and origin, have influenced consumer perceptions, behaviors, and preferences toward hen’s eggs^3^. It is also found that there are certain external and internal factors influence the characteristics of hen's eggs quality. For instance, external factors include egg oval shape that is clean and smooth, and strong and uniform shell color, while internal factors include yolk color, centrally held yolks that are firm and round, and a thick inner albumin layer^4^. Thus, in order to obtain high-quality hen's eggs, several recommendations should be followed in terms of appropriate diet, disease prevention, and appropriate storage time and temperature^4^.

### Egg quality classification

Hen’s eggs are classified into class A and class B based on certain quality characteristics. Class A Hen’s eggs are defined by their clean, undamaged, and normal shell and cuticle shape. Their air space should be no more than six millimeters. Their yolks are visible when candling and return to the eggs’ central position after a slight turn. This class has a clear and translucent white color and is free of germs and an unpleasant odor. Class B hen's eggs are those that do not conform to the Class A characteristics (EC, No. 589/2008)^5^.

The marketing standards for eggs stated by the Council Regulation (Commission Regulation) announced that it is preferably to store and transport eggs at constant temperature. Leaving cold eggs at room temperature may lead in water condensation on the outer surface of eggs and allow growth of bacteria in and on eggs (EC, No. 589/2008)^5^. It is advisable not to wash or clean eggs as this may cause damage to the egg shell and result in trans-shell contamination with bacteria. Ultra-violet rays are considered an alternative safety cleaning method (EC, No. 589/2008)^5^. Regarding the eggs expire date, it should not exceed 28 days after laying (EC, No. 589/2008)^5^. A Brazilian survey on food safety behavior during preparation, storage, consumption of hen’s eggs reported that 51.5% of the respondents read the label on the hen's eggs box, egg origin, and expire date. In the same study, 90.2% of people stored hen's eggs in the refrigerator, and 9.4% at room temperature^6^.

### Factors influencing egg quality and consumer habits

Consumer habits for specific hen's eggs characteristics vary from place to place. Many prefer farm hen's eggs than industrial hen's eggs as they think that they are more flavorful with better texture and color^7^.

Literature has reported abnormalities that can be attributed to hen’s age- when older hens lay eggs with bad quality- or may be due to certain diseases, changes in their hormones, certain medications, and stress^4^. Moreover, low level of calcium in the hens’ feed result in reduction in egg shell quality, egg size, shell thickness, and cracked shells^4^. Improper food handling practices as storing temperature, time, and cross contamination are associated with food borne disease^6^. Improper storage can accelerate bacterial growth, potentially compromising the integrity of the hen's eggs and increasing the likelihood of foodborne illnesses.

Hen's eggs borne human *Salmonellosis* can be explained by the contamination of the egg shell with *Salmonella* or its ability to gain entrance inside the egg during egg formation in hens’ utero^8^. When egg shells contaminated by fecal or environmental *Salmonella*, *Salmonella* can penetrate inside the egg through the pores present in the egg shell^8^. The risk of *Salmonella* enteritis in hen’s eggs increased in poor refrigeration, consumption of raw hen's eggs and time and temperature abuse^9^. As a result of salmonellosis, patients experience diarrhea, fever, abdominal pain or cramps, vomiting, headaches, and nausea. Incubation lasts between 8 and 72 h. Symptoms can last up to a week. Salmonella infections vary from mild to severe and are occasionally fatal. Other bacterial infections such as *E. coli* and Campylobacter are also possible health risks associated with hen’s eggs consumption, emphasizing the critical importance of proper handling, storage, and cooking to mitigate these health risks.

According to United States Department of Agriculture (USDA) regulations, only intact shell eggs are allowed to be marketed to consumers, while cracked eggs may be processed to dry egg products^10^. For shell egg cleanliness, USDA guideline requires shell eggs washing^8^.

Numerous studies have assessed consumers’ knowledge and practices related to the handling of chicken meat and eggs^2,3,6^. In the Palestinian context, published studies examining consumers’ knowledge and practices regarding hen’s eggs safety are scarce. Additionally, Palestine’s unique geopolitical situation and specific challenges related to food security and access may impact consumer perceptions of food safety. These factors are expected to play a critical role in shaping the understanding of hen’s eggs safety among consumers in Palestine, warranting a specific examination in this context. Thus, the purpose of this study was to determine participants' knowledge regarding hen's eggs safe purchase practices, storage, and consumption practices. It also aims to examine the differences in knowledge levels among various consumers’ groups and characteristics, as well as the relationship between knowledge levels and practices when it comes to hen’s eggs purchase, storage, and consumption. Understanding consumer behavior and knowledge gaps is vital for food safety and public health. Given the rising concern for foodborne illnesses, this study is essential for safeguarding the well-being of Palestinians. Raising awareness about hen’s eggs safety and proper handling can empower individuals to make informed choices and minimize the risk of diseases. Additionally, our research has practical implications for policymakers and regulatory agencies, facilitating the development of more effective guidelines to ensure hen’s eggs product quality and safety. This study serves as a key resource for implementing evidence-based strategies to enhance food safety and promote better hygiene practices for the Palestinian community.

## Methods

### Study design and settings

The current cross sectional online survey was conducted in West Bank, Gaza, and Jerusalem in Palestine. The study covered nine governorates: Nablus, Jenin, Tolkarem, and Qalqiliah in the north of West Bank; RamAllah, Jerusalem, and Bethlehem in the middle; Hebron in the south, and Gaza. The data were collected online using Google forms due to COVID-19 pandemic, and the link was shared on social media platforms such as well-known and large Facebook groups and popular WhatsApp groups. The participants were provided with a data collection link, which included an invitation statement outlining the study's objectives and emphasizing the voluntary nature of participation. Following this, participants were presented with an item asking if they were willing to participate in the survey, with response options of “Yes” or “No”. Those who selected “Yes” were subsequently directed to the subsequent sections. The data collection period lasted one month, started in May 2021 and completed in June 2021. The study protocol was approved by An-Najah National University’s IRB (Institutional Review Board) and the Ethical Committee, serial number is Ref: Agr.April.2021/1.

### Study population

This was a community based study. All Palestinian adults aged 18 years and above were eligible to participate in the study. The excluded participation criteria included: those did not click yes on the consent of participation, duplicated participation, were less than 18 years old and if any participant reported that he/she or any of his/her family member ‘*never buy or consume eggs’*.

### Sample size and sampling techniques

Sample size was estimated using G power software for sample size calculation; at alpha level of 0.05 with 5% margin of error and 80% power and 95% confidence level, and mean difference as the selected statistical test. The sampling method used in the study was voluntary sampling. In this research, voluntary sampling refers to a non-probability sampling technique where participants voluntarily choose to be part of the study without any random selection process. This sampling method was utilized due to certain practical constraints and the nature of the target population. Given the sensitive nature of the topic and the need to gain insights from willing participants, voluntary sampling was deemed the most feasible method to access respondents for our study.

### Data collection procedure

Participants in the study were asked to complete an online Arabic questionnaire. The questionnaire was divided into two sections; the first section contained sociodemographic variables such as age, gender, educational level, residence, economic and employment status, and marital status. The second section was allocated for the knowledge, practice, and observation of egg purchasing and consumption; a more detailed description of this division is provided in the next section.

### Data collection instruments

#### Questionnaire development

The questionnaire items were developed using data from the literature regarding hen's eggs safety behaviors, handling practices, preparation, and storage^3,6,11^, in addition to the European Commission's regulations and standards (EC) No. 589/2008^5^ and national regulations, and Palestinian Standards Institutions^12^. The research team developed the initial draft of the questionnaire, which consisted of four sections: Sect. “Introduction” contained four questions about hen’s eggs purchase, Sect. “Methods” contained fifteen questions on consumer knowledge of hen's eggs handling, storage, and preparation, Sect. “Results” contained fifteen questions on consumer practices of hen’s eggs handling, storage, and preparation, and Sect. “Discussion” contained six questions about consumer perceptions of hen’s eggs quality in local markets. Each section of the questionnaire was written in the participant's native language (i.e., the Arabic language). Five experts in assessment and food safety verified and approved the questionnaire's content validity. They recommended eliminating two items from the knowledge section and rewording three items in the practices section to improve clarity. All the reviewers’ suggestions and corrections were taken into account, and the final questionnaire was modified accordingly. A pilot study with 45 subjects was conducted to evaluate the questionnaire's internal reliability using SPSS™ version 21. Cronbach’s alpha coefficients were 0.85 and 0.75 for knowledge section and practices section respectively, indicating that the items had an acceptable level of internal consistency. The responses that were collected in the pilot study were subsequently eliminated from the analysis of the main study. The knowledge items were presented as statements, and subjects were required to indicate whether each statement was “true,” “false,” or “I don’t know.” The responses were coded as follows during the analysis phase: 1 point for the correct response and 0 point for the incorrect or “I don’t know” response. The sum of the answers was calculated to determine the subject’s overall score out of thirteen. Whereas the practices section consisted of fifteen items with three-point Likert scale: always, occasionally, or never.

### Data analysis

The final collected data were analysed based on the dichotomous Rasch model (i.e. knowledge with 0, 1) and polytomous Rasch model (i.e. practices with a 3-point scale) using Winsteps version 5.1.3^13^. The Rasch model for measurement was utilised because it enabled researchers to get the difficulty measure for each item and ability measure for each participant and place them on the same interval scale to see their distributions. The most difficult items to answer (knowledge) or endorse (practices) and most able participants are positioned toward the upper part of the scale and vice versa^13,14^. In addition, the statistical package for the social sciences SPSS version 21 used the Rasch output interval data files to conduct the inferential analyses (One-way analysis of variance (ANOVA) and Independent samples t-test) comparing mean scores of the knowledge construct across certain demographic variables (e.g., gender). All the results were displayed in Tables and Figures.

### Ethics approval and consent of participant

Ethical approval was obtained from the Institutional Review Board “IRB” at An-Najah National University in Nablus-Palestine and was performed in compliance with the Helsinki Declaration for research humans. Ref: Agr.April.2021/1

### Consent of participant

Informed consent was obtained from all participants before giving the required data.

## Results

This study aimed to determine Palestinian consumers’ knowledge level based on their responses to items on raw hen’s eggs, and to identify their practices in handling these hen’s eggs during purchase, preparation, storage and consumption. The Rasch model analyses showed the overall levels for knowledge and practices, and which knowledge items the participants got most correct and which practices items they highly endorsed. These results were followed by the inferential analysis using the interval data produced by the Rasch Model.

### Participants’ recruitment

A total of 620 participants responded to the online invitation and signed the online consent form. A total of 577 participants (88 Males and 489 Females) included in the final analysis because forty three participants were excluded due to their age (< 18 years old), never eat or buy hen's eggs, and missing data.

### Participants’ sociodemographic variables

Participants’ characteristics are summarized in Table 1. The mean age of the participants was: 32 ± 10.5 years, ranged from 18 to 63 years old. The participants were distributed according to their governorates; Nablus: 206 (35.7%), Jenin 43 (7.5%), Tulkarm 68 (11.8%), Qalqiliya 31 (5.4%), Ramallah 29 (5%), Bethlehem 12 (2.1%), Hebron 81 (14%), Jerusalem 47 (8.1%) and Gaza 60 (10.4%).

**Table 1:** Participants’ socio demographic characteristics.

Variables		n	%
Areas distribution	Northern areas	348	60.3
	Middle areas	88	15.3
	Southern area	81	14
	Gaza	60	10.4
Gender	Male	88	15.3
	Female	489	84.7
Marital status	Single	231	40
	Married	331	57.4
	Others	15	2.6
Educational level	No formal education	1	0.2
	Primary education	5	0.9
	Secondary education	52	9
	Diploma	35	6.1
	Degree	413	71.6
	Postgraduate	71	12.3
Work status	Governmental officer	79	13.7
	Private work- office	101	17.5
	Business s	17	2.9
	Technician	9	1.6
	Employee	7	1.2
	Not working	364	63.1
Income NIS/ month	< 1500	68	11.8
	1500–3000	187	32.4
	3001–5000	153	26.5
	More than 5000	145	25.1

### Participants’ knowledge of hen's eggs consumption safety

**Figure 1:**
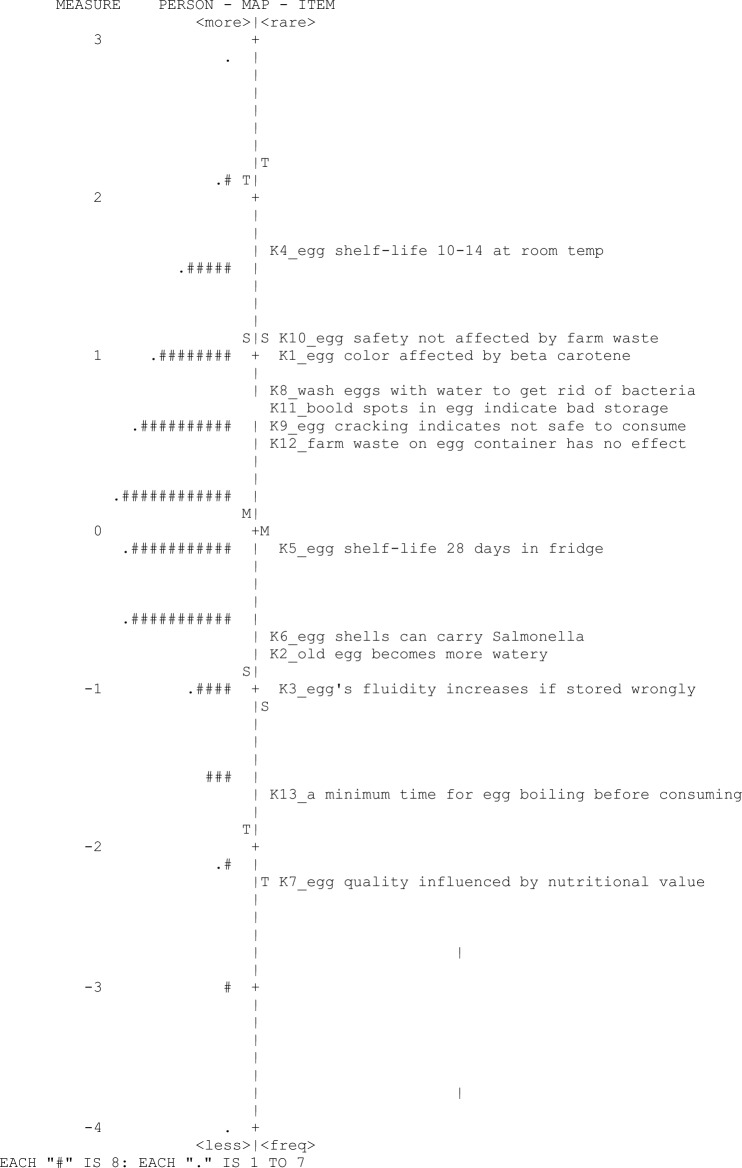
Wright item map—Knowledge.

The Rasch analysis showed that on average, the participants were marginally/slightly more able than the item difficulty since the person ability mean was 0.10 logit, higher than the mean of item difficulty 0.0 logit as shown in Fig. 1 and Table 2, indicating quite low level of knowledge of hen's eggs consumption safety hen’s eggs. More specifically, the item map (Fig. 1) shows that the person ability measures spanned about 7.29 logits (from − 4.37 to + 2.92) while item difficulty measures spread was about 3.89 logits (from − 2.24 to + 1.65). It also clearly displays the distribution/hierarchy of all items on one interval scale. The mostly corrected items are placed towards the lower part, and the least corrected ones are placed towards the upper part of the scale. The most difficult items for the participants to answer were item 4 *(The shelf-life of the hen's eggs is 10–14 days at room temperature*, 1.64 logit); item 10 (*The safety of hen’s eggs consumption is not affected by the presence of farm wastes on the shells,*1.06 logit) ; item 1(*The color of the hen's eggs yolk is affected by the beta carotene content, and the yellow color increases when beta-carotene increases*, 0.98 logit); item 8 (*It is recommended to wash hen’s eggs with water before using it to get rid of bacteria on their shells, 0.80*); item 11 (*The presence of blood spots inside the hen’s eggs means bad storage conditions*, 0.73 logit); item 9 (*The presence of cracking on the eggshells indication that the hen’s eggs are not fit for consume*, 0. 62 logits), and item 12 (*The present of farms waste on the hen’s eggs containers has no effect on* hen’s egg *safety*, 0.56 logits). On the other hand, the easiest items to answer were item 7 (*The quality of the hen’s eggs is influenced in terms of nutritional value by the way chickens are raised (regular or free-range farm) − *2.23 logit); item 13 (*There is a minimum boiling time for hen’s eggs, to assure the safety before consuming the boiled eggs*., -1.68 logit); item 3 (*An egg's fluidity increases if it’s stored in wrong way*,− 0.99 logit); item 2 (*The old hen's eggs becomes more watery*, − 0.77 logit); item 6 (*The hen’s eggs shells can carry the Salmonella (pathogenic bacteria)*, − 0.63 logit; and item 5 (*The shelf-life of the hen’s eggs inside the fridge is 28 days from the day of the egg laying*, − 0.12 logit.

**Table 2:** Difficulty measures of knowledge items.

No	Item	Measure	S.E
4	The shelf-life of the hen's eggs is 10–14 days at room temperature	1.65	0.11
10	The safety of hen's eggs consumption is not affected by the presence of farm wastes on the shells	1.06	0.10
1	The color of the hen's eggs yolk is affected by the beta carotene content, and the yellow color increases when beta-carotene increases	0.98	0.10
8	It is recommended to wash hen's eggs with water before using them to get rid of bacteria on their shells	0.80	0.09
11	The presence of blood spots inside the hen's eggs means bad storage conditions	0.73	0.09
9	The presence of cracking on the eggshells indicates that the eggs are not fit for consume	0.62	0.09
12	The present of farms waste on the hen's eggs containers has no effect on egg safety	0.56	0.09
5	The shelf-life of the hen’s eggs inside the fridge is 28 days from the day of the egg laying	− 0.12	0.09
6	The hen's eggs shells can carry Salmonella (pathogenic bacteria)	− 0.63	0.10
2	The old hen's eggs become more watery	− 0.75	0.10
3	An egg's fluidity increases if it’s stored in a wrong way	− 0.99	0.10
13	There is a minimum boiling time for eggs to assure the safety before consuming the boiled hen's eggs	− 1.68	0.12
7	The quality of the hen's eggs is influenced in terms of nutritional value by the way chickens are raised (regular or free-range farm)	− 2.23	0.14

### Practices

**Figure 2:**
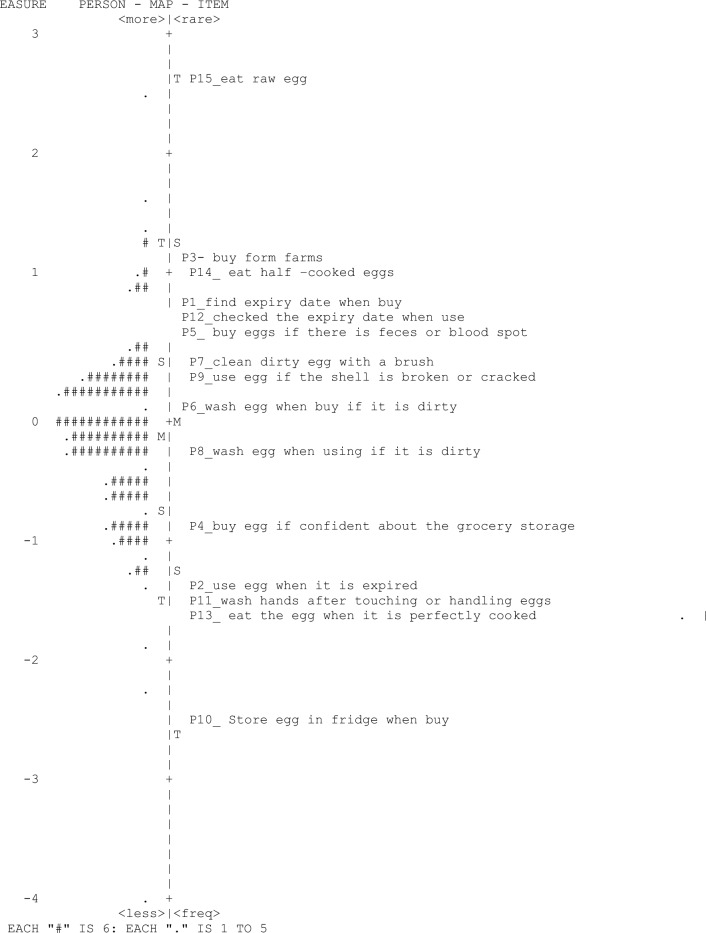
Wright item map—practices.

Overall, the Rasch analysis revealed that it was not easy for the participants to endorse or agree on the items pertaining their practices in handling the raw hen's eggs during purchase, preparation, storage and consumption as shown in (Fig. 2 and Table 3). The Mean of participants’ ability was (− 0.11 logit), lower than the Mean of item difficulty (0.0 logit). The Item-Map (Fig. 2) shows that the most difficult item to be endorsed by the participants was item 15 (*I eat raw hen’s eggs,* 2.68 logits); followed by item 3 (*I buy hen’s eggs from farms only*, 1.08 logit); item 14 (*I eat half-cooked (i.e. still semi liquid) hen’s eggs*, 0.94 logits); item 1 (*I buy hen’s eggs only if the expiry date is printed on the hen’s eggs box*, 0.80 logits); item 12 (*I check the expiry date of hen's eggs when I use*, 0.78 logits); item 5 (*I buy the hen’s eggs if there is chicken feces or blood spot on the eggshells)*, 0.76 logits); item 7 (*I clean hen’s eggs with a brush if they have obvious dirty on the shells*, 0.49 logits); item 9 (*I used the hen’s eggs if the shell is broken or cracked,* 0.34 logits); and item 6 (*I wash the hen’s eggs when I buy it if it are dirty*, 0.10 logits). On the other hand, the easiest items to be endorsed by the participants were items 10 (*I store the hen’s eggs in the fridge when I buy*, − 2.45 logit); item 11 (*I wash my hands after I touch or handle the hen’s egg*, − 1.56 logits); item 13 (*When you eat the hen’s egg, you eat it after it is perfectly cooked (solid).*, − 1.45 logit); item 2 (*I don’t use hen’s eggs when it is expired.*, − 1.36 logit); item 4 (*I buy the hen's eggs only if I confident about the grocery and storage temperature*, − 0.89 logits); and item 8 (*I wash the hen’s eggs just before using it if it is dirty*, − 0.27 logits).

**Table 3:** Participants’ hen’s egg safe handling practices.

No	Item	Measure	S.E
15	I eat raw hen’s eggs	2.68	0.14
3	I buy hen’s eggs from farms only	1.08	0.07
14	I eat half-cooked (i.e. still semi liquid) hen’s eggs	0.94	0.17
1	I buy hen’s eggs only if the expiry date is printed on the egg box	0.80	0.06
12	I check the expiry date of hen's eggs when I use	0.78	0.06
5	I buy the hen’s eggs if there is chicken feces or blood spot on the eggshells	0.76	0.06
7	I clean hen’s eggs with a brush if they have obvious dirt on the shells	0.49	0.06
9	I use the hen’s eggs if the shell is broken or cracked	0.34	0.06
6	I wash the hen’s eggs when I buy them if they are dirty	0.10	0.06
8	I wash the hen's eggs just before using them if they are dirty	− 0.27	0.06
4	I buy the hen's eggs only if I confident about the grocery and storage temperature	− 0.89	0.06
2	I don’t use hen’s eggs when they are expired	− 1.36	0.07
13	I eat perfectly cooked (solid) hen’s eggs	− 1.45	0.07
11	I wash my hands after I touch or handle the hen's eggs	− 1.56	0.08
10	I store the hen's eggs in the fridge when I buy them	− 2.45	0.08

It seems that the participants are not so concerned about checking the expiry dates or dirtiness of the hen’s eggs when they purchase hen’s eggs. However, they were very particular about being clean when preparing or cooking hen’s eggs and the place where they buy the hen's eggs and proper storage.

### Relationship between consumers’ knowledge of hen’s egg safety with selected variables

#### The relationship between consumers’ knowledge and sociodemographic variables

The sociodemographic variables and their effect on consumers’ knowledge were reported in Table 4. The results demonstrated that there were no statistically different effects according to the areas, marital status, educational level, work status and income in the consumers’ knowledge and practices. Whereas, participants gender showed significant differences *p* < 0.05 in terms of knowledge scores, where females had significantly high knowledge scores as compared to males’, *p* < 0.05 (Table 4).

**Table 4:** Participants’ knowledge according to their sociodemographic distribution presented in mean ± sd.

Variables		n	Mean ± sd	*P* value
Areas distribution	Northern areas	348	0.11 ± 1.0	0.750^1^
	Middle areas	88	0.55 ± 1.1	
	Southern area	81	0.11 ± 1.2	
	Gaza	60	0.15 ± 0.9	
Gender	Male	88	− 0.18 ± 1.1	0.006*^2^
	Female	489	0.15 ± 0.99	
Marital status	Single	231	0.11 ± 1.1	0.668^1^
	Married	331	0.10 ± 0.96	
	Others	15	− 0.15 ± 1.2	
Educational level	School education	58	1.04 ± 0.45	0.137^1^
	Diploma	35	− 0.04 ± 1.1	
	Degree	413	0.13 ± 0.92	
	Postgraduate	71	0.13 ± 0.92	
Work status	Governmental officer	79	− 0.05 ± 1.0	0.627^1^
	Private work- office	101	0.21 ± 0.87	
	Business s	17	0.05 ± 0.61	
	Technician	9	0.14 ± 1.8	
	Employee	7	0.015 ± 0.39	
	Not working	364	0.11 ± 1.1	
Income NIS/ month	< 1500	68	− 0.01 ± 0.99	0.470^1^
	1500–3000	187	0.17 ± 1.1	
	3001–5000	153	0.079 ± 0.97	
	More than 5000	145	0.03 ± 0.97	

*Significant, *p* < 0.05 using Independent sample t- test. ^1^one Way Anova, ^2^Independent sample t-test.

#### The relationship between participants’ knowledge and hen's egg purchase variables

For the hen’s eggs purchase practices, Table 5 shows there were no significant differences in knowledge scores for the item related to involvement in buying hen’s egg. However, the participants who usually buy the hen’s eggs for their family scored lower than participants who sometimes do or they don’t do. Moreover, consumers who purchased hen's eggs had higher knowledge regarding hen's eggs quality and safety.

**Table 5:** Participants’ knowledge according to hen's egg purchase practices.

Variables		n	mean ± sd	*P* value
Do you usually buy the hen's egg for your house?	Yes I usually do it by myself	85	0.07 ± 0.94	0.964
	I do sometimes	146	0.13 ± 1.05	
	No I don’t buy the egg, family member do that	319	0.09 ± 1.02	
	Get the egg from chicken farm	27	0.12 ± 1.08	
Source of the hen’s egg?	Neighboring groceries	368	0.13 ± 1.03	0.747
	Neighboring farms	21	0.06 ± 0.92	
	Hypermarket	161	0.05 ± 1.0	
	Directly from producers	27	0.047 ± 1.02	
Amount of hen’s eggs purchased	< 10 eggs/time	43	0.15 ± 0.99	0.241
	10–20 eggs/time	180	− 0.03 ± 1.03	
	20–30 eggs/time	354	-0.16 ± 1.4	

*significant *p* < 0.05 using one way ANOVA test.

#### The relationship between consumers’ knowledge and practices variables

Table 6 illustrates the relationship between consumers’ knowledge and practices. The results revealed there a was significant difference *p* < 0.05 in terms of knowledge scores with the item (*I clean the hen’s eggs with a brush if they have obvious dirt on the shells*); participants *who usually do* or *sometimes do* scored higher scores as compared to participants who *do not do*.

**Table 6:** Participants’ knowledge according to practice.

Item no	Variables		n	Mean ± sd	*P* value
1	I find the expiry date on the hen’s egg box when I buy it	Always	67	0.08 ± 1.0	0.714
		Sometimes	179	0.04 ± 1.1	
		Never	331	0.14 ± 0.97	
2	I don’t use hen’s eggs when it is expired	Always	414	0.09 ± 1.05	0.099
		Sometimes	78	-0.07 ± 1.02	
		Never	78	0.27 ± 0.78	
3	I buy hen’s eggs from farms only	Always	24	-0.1 ± 0.82	0.475
		Sometimes	199	0.13 ± 0.95	
		Never	350	0.10 ± 1.07	
4	I buy the hen’s eggs only if I confident about the grocery and storage temperature	Always	309	0.104 ± 1.01	0.555
		Sometimes	184	0.13 ± 1.04	
		Never	80	-0.01 ± 0.99	
5	I buy the hen’s eggs if there is chicken feces or blood spot on the eggshells	Always	44	0.13 ± 1.3	0.766
		Sometimes	233	0.07 ± 0.93	
		Never	298	0.11 ± 1.05	
6	I wash the hen’s eggs when I buy it if it are dirty	Always	167	0.06 ± 0.97	0.739
		Sometimes	177	0.08 ± 1.01	
		Never	232	0.14 ± 1.06	
7	I clean hen’s eggs with a brush if they have obvious dirty on the shells	Always	110	0.15 ± 0.94	0.005*
		Sometimes	175	0.28 ± 0.98	
		Never	290	-0.04 ± 1.05	
8	I wash the hen’s eggs just before using it if it is dirty	Always	234	0.07 ± 0.86	0.053
		Sometimes	158	0.26 ± 1.03	
		Never	182	-0.001 ± 1.12	
9	I used the hen’s eggs if the shell is broken or cracked?	Always	113	-0.05 ± 1.04	0.141
		Sometimes	212	0.14 ± 0.98	
		Never	248	0.15 ± 1.04	
10	I store the hen’s eggs in the fridge when, I bought it?	Always	505	0.14 ± 0.99	0.042*
		Sometimes	53	-0.04 ± 1.1	
		Never	17	-0.43 ± 1.2	
11	I wash my hands after, I touch or handle the hen’s egg?	Always	422	0.13 ± 1.01	0.217
		Sometimes	104	0.06 ± 1.08	
		Never	47	-0.12 ± 1.02	
12	I check the expiry date of hen’s eggs when, I bought it?	Always	65	0.16 ± 1.02	0.527
		Sometimes	189	0.038 ± 1.03	
		Never	322	0.133 ± 1.00	
13	When you eat the hen’s egg, you eat it after it is perfectly cooked (solid)	Always	406	0.12 ± 0.98	0.478
		Sometimes	11	0.10 ± 1.07	
		Never	51	-0.07 ± 1.17	
14	When you eat the hen’s egg, you eat it after it is half-cooked (still semi liquid)	Always	63	0.03 ± 0.92	0.329
		Sometimes	149	0.00 ± 1.11	
		Never	355	0.156 ± 0.99	
15	When you eat the hen's egg, you eat it raw?	Always	10	0.25 ± 0.82	0.061
		Sometimes	34	-0.29 ± 1.01	
		Never	524	0.12 ± 1.01	

*significant *p* < 0.05 using one way ANOVA test.

## Discussion

The current study was performed originally to assess consumers’ knowledge and practices regarding hen’s eggs handling practices during purchase, preparation, storage and consumption, and to verify whether consumers’ knowledge is correlated with their practices. Based on the current literature, this is the first research of this kind conducted in Palestine. Therefore it provides essential information to propose other interventional and educational programs in this field.

### Knowledge about hen’s egg safety

The reported results showed that only 32.8% of the participants knew that the hen’s egg yolk color changes according to the concentration of beta-carotene in their feeds. Roberts (2019) reported that variation in yolk color is due to the source of the pigmentation (natural or synthetic) and their levels of utilization and combinations between xanthophylls, stability and availability of xanthophylls, feed composition, stress, genetics, health status, as well as other factors^4^. This lack of knowledge potentially indicates a limited awareness of the essential nutrients present in hen’s eggs and their potential health benefits. Most respondents (67.6%) declared that old hen’s eggs become more watery than fresh hen’s eggs. Moreover, the highest participants (71.8%) declared that hen’s egg fluidity increases when it is stored in a wrong way. This reflects that most of the participants used to cook or utilize hen’s eggs. A total of 40.7% of participants were aware that hen’s eggs last 10–14 days at room temperature, while 55.1% knew their shelf-life is up to 28 days from laying. Only 65 participants routinely checked hen's egg expiry dates. Moreover, 65.3% recognized the risk of Salmonella on eggshells, and 87.9% believed hen's egg quality depends on the hen's diet. Additionally, 36.5% recommended washing hen’s eggs before use. The Food Agriculture Organization Food and Agriculture Organization (FAO) conducted a survey with Palestinian consumers to determine their knowledge and practices related to the consumption of hen’s eggs and showed that 48.4% respondents wash hen's eggs before cracking hen’s eggs shells^15^. In the current study, only 39.9% of respondents believed that the presence of cracks on eggshells indicates that eggs are not fit for consumption. It is crucial to handle hen’s eggs with care and to discard those with extensive cracks, minor hairline cracks do not necessarily render the hen's eggs unsafe for consumption if they are used promptly and properly cooked. Moreover, 31.2% and 41.1% of the participants considered the presence of farm wastes on the hen’s egg shells and egg box cartons do not affect hen’s eggs safety, respectively. Farm waste poses a serious risk to food safety because it can include dangerous bacteria and diseases that can infect eggs. Ignoring the possible effects of farm waste on the safety of hen's eggs could result in incorrect handling procedures, which could expose consumers to foodborne illnesses. Promoting a thorough awareness of the value of hygiene measures in guaranteeing the safety and quality of hen's eggs requires educating customers about the significance of maintaining cleanliness throughout the hen's egg production and distribution process. In order to lower the risk of foodborne illnesses and provide a safer environment for food consumption, it is important to emphasize the importance of good sanitation practices on farms and during packaging.

Surprisingly, just 37.6% of participants knew that blood spots in hen’s eggs suggest poor storage. However, 82% were aware of the necessary boiling time to make hen's eggs safe to eat. The survey showed that awareness of hen's egg safety is lower than anticipated, raising questions about the causes of this knowledge gap. This finding goes with^16^ who found out that in Australia there was a lack of knowledge surrounding the risks of handling and using hen's eggs collected from domestic poultry.

Gender differences are found in various studies. For instance, a study conducted in the United States of young adults (mean age 19.9) demonstrated that females significantly identified safer food handling practices compared to males^17^. The gender difference in hand washing behavior when handling raw hen’s eggs is particularly interesting, since several previous studies have reported that women wash their hands differently from men^18,19^; indicating that food preparation is a task to be conducted by females in the household. Other studies conducted in Korea and Thailand, surveying 100 participants from each country, investigated hen's egg purchase, storage, and preparation practices. They discovered that female respondents were more likely to wash hen's eggs using soap compared to their male counterparts^20^.

### Storage and handling practices

In this study, several noteworthy findings emerged concerning participants’ hen’s egg purchasing habits and their corresponding levels of knowledge about hen’s egg quality and safety. The study found that regular hen's egg buyers for their families scored slightly lower on knowledge about hen’s egg quality and safety than those who buy hen’s eggs less often or not at all, although the difference was not statistically significant. These results were reported by^20^. Increasing knowledge regarding feeding and methods of production significantly affect hen’s egg purchase^3^. The hen's egg source does not affect consumers’ knowledge. The majority of respondents (368) answered that they buy hen's eggs from neighboring groceries. This finding was similar to the results obtained by Hessel et al., (2019), where 95.3% of respondents bought hen's eggs from local markets and greengrocers. However, 354 participants buy 20–30 hen’s eggs each time, implying that they highly consume hen’s eggs^6^.

According to the findings of the present study, 331 participants never check the expiry date on hen’s eggs when they purchase; while only 67 individuals locate the expiry date on the hen’s egg box. It is important to mention that according to the mandatory technical instruction of hen's egg marketing number 30–2011 by Palestinian Standard Institution (PSI), it is recommended that each hen’s egg box should display the expiry date^12^. Regarding the question “*I don't use hen’s eggs when it is expired*” 414 responded as *always*. The lack of small hen’s egg producers and difficulty in locating producers in some cities may explain why 350 participants never buy hen’s eggs directly from the producers. 309 participants buy hen’s eggs when they are confident about the grocery and the storage temperature. It is observed that in Palestine as well as many other countries such as India, hen’s eggs are sold at room temperature because local grocery stores do not have enough cold storage facilities^21^.

Only 298 respondents said that they never buy hen’s eggs if there are chicken feces or blood spots on the eggshell; while 233 said sometimes buy hen’s eggs with the presence of chicken feces or blood spots on the shell. This indicates that the consumers’ knowledge regarding the safety is low. One recommendation by PSI asserts that raw hen’s eggs should be free from any abnormalities, chicken residue or blood spot^4,12^ Regarding the washing hen’s eggs, 167 participants always wash eggs if they are dirty, 177 sometimes wash eggs, while 232 never do that. As stated in the Commission Regulation (EC) No 589/2008^5^, it is not recommended to wash Class A eggs due to the potential damage to the physical barriers, including cuticle, caused by washing^5^. Furthermore, eggs that are dirty may contain harmful bacteria that can enter eggs; and if they are not cooked properly, they can potentially cause food poisoning. Cracked eggs increase the risk of food poisoning^22^. Dirty eggs should not be sold for raw consumption but can be used for processing or animal feed. The USDA recommends not washing raw eggs to prevent cross-contamination and foodborne illnesses^23^. Many food safety guidelines mention that the inner contents of the hen's egg can shrink if the wash water is unsuitable. As a result, wash water and pathogens might be drawn into the egg through the shell pores^8^. Washing dirty hen's eggs can burden egg washing systems and, if done, should follow best practices: wash immediately after collection, discard very dirty eggs, use water at 41–44 °C with a pH ≥ 10.5 for sanitizers, avoid soaking during washing, and always dry eggs afterwards to reduce the risk of microbial ingress^22,24^.

### Practices in cleaning and washing hen's eggs

The study found a significant relationship (*p* < 0.05) between participant knowledge and the practice of cleaning hen’s eggs with a brush, with most (290) never or sometimes using this method, potentially indicating a lack of awareness about effective hen’s egg cleaning techniques such as brushing, which can remove contaminants and reduce hazards. It is interesting to report that a big number wash dirty hen’s eggs before use as recommended by^15^ FAO 2017 to clean eggs shells with soap and water before cooking them^25^.

The survey revealed that while 248 participants safely never use broken or cracked hen’s eggs, 325 do use them always or sometimes, reflecting inadequate knowledge on proper hen’s egg usage practices. This lack of awareness could explain why consumers often find such hen's eggs in purchased cartons, differing from an Australian study where only three participants used cracked hen’s eggs^16^. The broken or cracked hen’s eggs should be excluded from the uncracked eggshells. Hen’s eggs that have cracked shells are extremely prone to contamination by bacteria, thereby reducing the quality and posing health hazards to consumers. Cracked or broken hen’s eggs should not be sold^26^. In addition, bacteria can enter hen’s eggs contents when they are broken^24^.

A considerable number of respondents (505) store hen's eggs at home in their refrigerators, showing a correct safety practice. To prevent the growth of pathogenic bacteria such as *Salmonella*, spp present in hen’s eggs, eggs can be stored below 8 °C^5,15^. Recently, Sabbah and Al-Asmar^27^, evaluated the quality and safety of the raw hen’s eggs in the north Palestinian market, and they concluded that *Escherichia coli* contamination was detected on eggshells sourced from the Jenin governorate. Concurrently, eggshells from the Tulkarm governorate were found to be contaminated with *Salmonella enteritidis*. This study conclusively demonstrates the absence of essential sanitary practices such as grading, cleaning, and cooling prior to distribution. This neglect poses a potential microbiological health risk to consumers.

The absence of cold storage facilities in local grocery stores is a significant factor that impacts hen’s egg safety in the Palestinian context. Hen’s eggs storage may be expose to risk in the absence of sufficient refrigeration, which raises the possibility of bacterial growth and contamination. Of the survey respondents, 422 always wash their hands after handling hen's eggs, reducing the spread of potential microbes from the egg surfaces to the kitchen or ready-to-eat foods. Shell contamination may also stem from nesting materials, dust, feed, containers, and contact with humans and animals^6^.

Challenges and local climate may increase the risk of bacterial contamination and spoilage of hen's eggs. A small proportion of participants (65) always check hen’s egg expiry dates, while many (322) never do, indicating limited awareness of food shelf-life. Additionally, most participants (422) eat well-cooked hen's eggs, whereas fewer (63) eat them half-cooked, and only a few (10) consume them raw. The survey conducted by FAO showed that 76.6% respondents adhered to correct practices—i.e., they cook the hen's egg until the yolk is completely solid. As mentioned by ^23^ that no one should eat foods containing raw hen's eggs. Pasteurized hen's eggs can, however, be eaten without cooking.

Based on the findings, to improve hen's egg safety, local stores must prioritize cold storage to lessen contamination risks, and stringent quality control must be enforced throughout hen's egg production and distribution. To ensure safer hen's eggs consumption, education campaigns need to highlight proper handling, storage, and cooking. Practical tips can help consumers make informed choices and foster better hygiene practices. Collaborative efforts from government, community, and food industry stakeholders are essential to promote consumer education and enforce safety measures for improved public health in Palestine.

## Conclusion

The survey results show participants lack knowledge about hen’s egg safety practices. They easily understood the nutritional value and cooking time for hen's eggs but struggled with safety-related questions. Storing hen’s eggs in the fridge was simple for them, but eating raw hen's eggs was challenging. There were no major differences in hen's egg safety knowledge across sociodemographic groups, except between genders. The study identified the relationship between consumer’s knowledge and practices variables showed significant differences *p* < 0.05; different knowledge regarding the cleaning practices of hen's eggs, eating the raw hen’s eggs without good handling or suitable heat treatment. The most participants involved in this survey bought about 20–30 hen’s eggs each time from the neighboring groceries. Many participants used cracked or dirty hen's eggs, against national and international standards. Unsafe practices, like storing dirty hen's eggs with ready-to-eat foods, risk cross-contamination. The study found significant discrepancies between actual hen's egg handling, purchasing, and storage practices and the safety guidelines set by PSI, FDA, and USDA. Hen's eggs can harbor dangerous pathogens like *Salmonella*, which can cause serious illness or even be fatal, particularly for vulnerable populations such as the elderly, infants, pregnant women, and immunocompromised individuals. Understanding where the knowledge gaps and unsafe practices exist can lead to targeted education and interventions to prevent outbreaks. It is recommended to increase the consumer awareness regarding the best method for purchasing, storage and handling the hen's eggs. Wherefore, Arabic and English booklet and public seminars was prepared by the authors of this article to share with people in order to contribute with the government to rise the public awareness regarding the best practices of using the hen's eggs. The booklet will cover a variety of issues, including how to handle and store hen's eggs properly, recognize safe and fresh egg indicators, recognize the nutritional value of hen's eggs, and use good hygienic practices when preparing and cooking hen’s eggs.

The study’s strengths include a sufficient sample size of 577 respondents, a thorough questionnaire covering a variety of hen’s egg handling topics, and a rigorous approach utilizing the Rach and Rasch measurement models. The gender-based study offers insightful information, and its applicability to policymakers and health authorities is highlighted by its practical ramifications. The study’s shortcomings, however, are its dependence on self-reported data, limited geographic emphasis, and possible selection bias resulting from the voluntary sampling especially for the online survey. In addition the percentage of women participated in the study exceeded the men participation. However, it is anticipated that this would be the case, as in Palestine, the majority of food purchase and preparation responsibilities are typically assumed by women. Furthermore, women tend to exhibit greater interest in matters related to nutrition.

By recognizing these shortcomings and enhancing their positive aspects, the conclusions on the methods used by Palestinian customers to handle hen's eggs can be reinforced even more. In order to facilitate future research and gain a thorough grasp of Palestinian consumers' knowledge and practices about handling raw hen's eggs, we recommend that proactive consumer education be implemented, taking into account contextual and cultural factors. Let us work together to promote a food environment that is safer and healthier, where each egg on the table represents our dedication to the prosperity and well-being of our communities in Palestine.

## Data Availability

The dataset used and analyzed in this study is available from corresponding Author on reasonable request.
